# Enhancing percutaneous kyphoplasty efficacy in elderly osteoporotic fractures through optimal cement filling ratio

**DOI:** 10.3389/fendo.2024.1359550

**Published:** 2024-05-10

**Authors:** Ningxue Sun, Yu Zhang, Deqian Xie, Yating Chen, Yang Liu

**Affiliations:** ^1^ Department of Spinal Surgery, The First Affiliated Hospital of Dalian Medical University, Dalian, Liaoning, China; ^2^ Department of Urology, The First Affiliated Hospital of Dalian Medical University, Dalian, Liaoning, China; ^3^ Department of Radiology, The First Affiliated Hospital of Dalian Medical University, Dalian, Liaoning, China

**Keywords:** percutaneous kyphoplasty, osteoporotic vertebral compression fracture, bone cement filling ratio, osteoporosis, bone cement

## Abstract

**Objective:**

To explore the appropriate bone cement filling ratio in percutaneous kyphoplasty (PKP) for the treatment of osteoporotic vertebral compression fractures (OVCF).

**Methods:**

Clinical and radiological data from 150 OVCF patients treated with PKP were retrospectively analyzed. Patients were categorized into three groups based on bone cement filling ratio: low (<0.4), medium (0.4-0.6), and high (>0.6) filling ratio groups. The clinical characteristics (age, gender, BMI, etc.) and related study data (bone cement leakage and its location, pre/post-operative Visual Analogue Scale (VAS), pre/post-operative Oswestry Disability Index (ODI), vertebral height restoration, kyphotic Cobb angle, etc.) among the three groups were compared using statistical software to compare to identify the most appropriate cement filling ratio.

**Results:**

The 0.4-0.6 group presented a lower cement leakage rate compared to the >0.6 group, and there were no significant differences in pre-operative VAS, post-operative day 2 VAS, post-operative month 1 VAS, and pre-operative ODI (p>0.05). However, significant differences were observed in post-operative month 3 VAS (p=0.002), post-operative day 2 ODI (p=0.002), post-operative month 1 ODI (p<0.001), and post-operative month 3 ODI (p<0.001). The “0.4-0.6” group showed better pain improvement and functional recovery compared with the “>0.6” group at the 3-month follow-up. While presenting the best vertebral height restoration, the “>0.6” group also exhibited the greatest variability. Additionally, no significant difference in Cobb angle changes was observed among the groups.

**Conclusion:**

A bone cement filling ratio of 0.4-0.6 in PKP treatment for OVCF strikes a favorable balance between complication reduction and positive patient outcomes, warranting it as an optimal filling volume.

## 1 Introduction

Osteoporosis is a systemic disease caused by reduced bone density and bone mass (1). Vertebral compression fractures are one of the most common complications of osteoporosis, known as osteoporotic vertebral compression fractures (OVCF). OVCF is prevalent in the elderly population, with age being an independent risk factor accounting for its occurrence (2). With the aging population in China, the incidence of this disease is on a yearly rise, which is notably observed among elderly female patients, particularly postmenopausal women with osteoporosis related to estrogen deficiency (3, 4). Clinically, OVCF is primarily associated with decreased mobility, an increased risk of bed-related complications, and a higher mortality rate (5).

The treatment goals for patients with OVCF are to restore mobility, alleviate pain, and prevent new fractures. Traditional conservative treatments include bed rest, opioid analgesics, and external fixation supports to relieve pain and strengthen the vertebrae (6, 7). However, prolonged bed rest tends to lead to various complications, such as pneumonia, bedsores, and deep vein thrombosis. Additionally, patients with OVCF under conservative treatment are exposed to prolonged pain and increased bone demineralization, which in turn raises the risk of further progression or recurrence of vertebral fractures (8). To this end, surgical treatment is often chosen for patients with OVCF. Currently, percutaneous vertebroplasty (PVP) and percutaneous kyphoplasty (PKP) are effective surgical methods for treating OVCF. Both procedures can effectively alleviate pain in patients with OVCF and restore their vertebral height (9). Alleviation of pain and restoration of vertebral height are reliable indicators for assessing the efficacy of bone cement therapy. Currently, compared to PVP, PKP allows for a larger volume of bone cement injection and facilitates vertebral height restoration through balloon expansion, leading to rapid pain relief for patients (10).

However, existing studies still demonstrate the limitations of PKP treatment, including bone cement leakage, unsatisfactory repositioning, and postoperative complications (such as fractures of adjacent vertebrae and re-fracture of the treated vertebra). In the case of insufficient amount of bone cement filling, the improvement in symptoms of the patient’s injured vertebra is not optimal, and the probability of re-fracture increases significantly. However, excessive filling of bone cement can lead to over-strengthening of the injured vertebra, thereby increasing the risk of bone cement leakage and fractures in adjacent vertebrae (11). Bone cement leakage into spinal or nerve root canals can cause neurological complications like paralysis and nerve compression. If the cement enters blood vessels, it can result in a life-threatening pulmonary embolism (12). At the same time, bone cement leakage into the intervertebral disc accelerates disc degeneration, causing the disc to lose its cushioning function. This abnormal load transmission increases the stress on adjacent vertebrae, potentially leading to fractures in the neighboring vertebrae (13). Hence, the present research group advocates for controlling the amount of bone cement used during filling to a certain extent. This measure can potentially decrease unnecessary postoperative complications, thereby enhancing the patient’s quality of life.

This study aims to investigate the relationship between the volume of bone cement injection and vertebral body volume through a retrospective analysis of case data. It also aims to observe the prognosis of patients with different bone cement filling ratios postoperatively. By balancing clinical efficacy and postoperative complications, the study intends to determine the optimal intraoperative bone cement injection percentage, thereby providing individualized treatment plans for patients with osteoporotic vertebral compression fractures. Consequently, postoperative complications can be potentially avoided, and clinical outcomes can be improved, further guiding and developing clinical strategies for OVCF patients, and ultimately better benefiting future OVCF patients.

## 2 Materials and methods

### 2.1 General information

**Figure 1:**
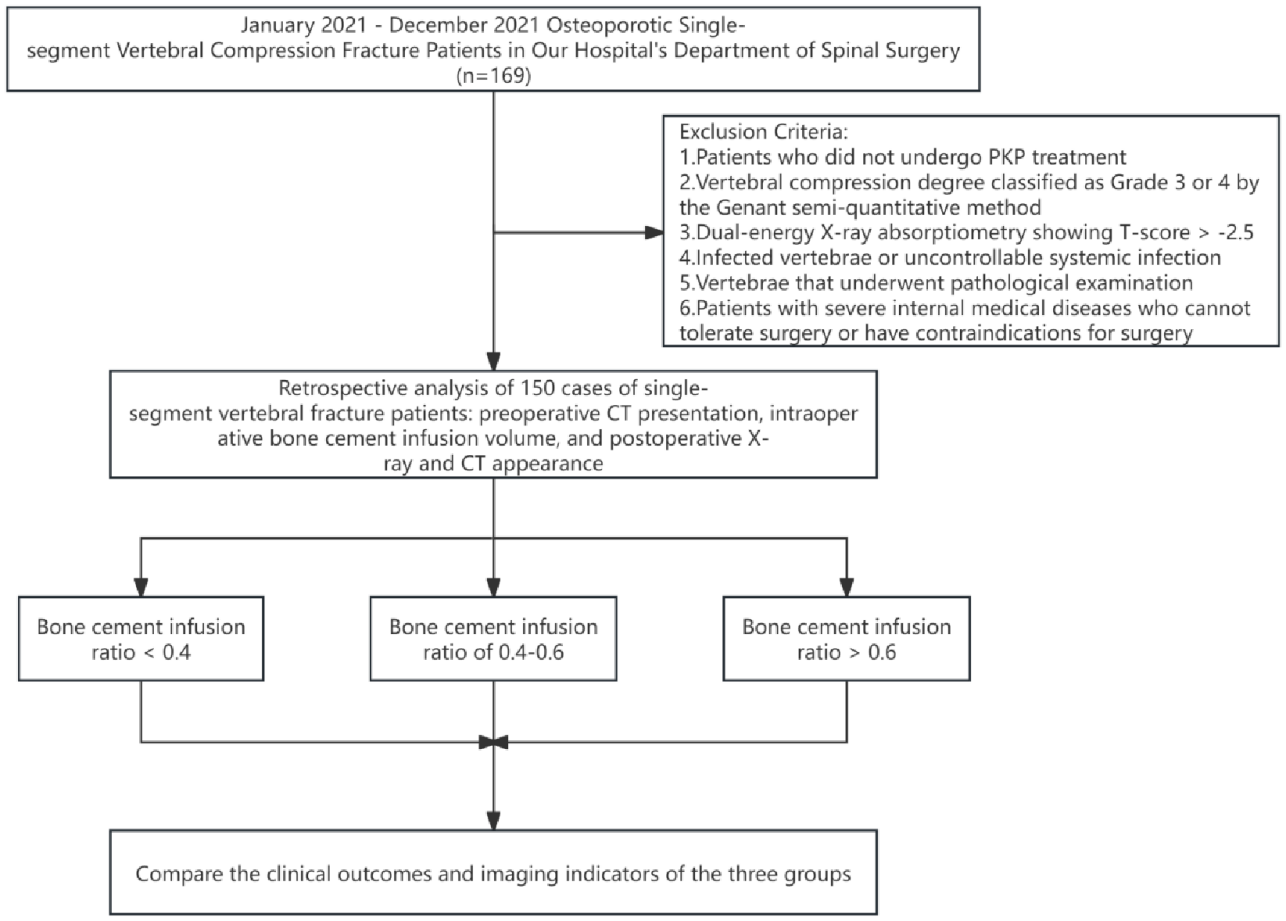
A flow diagram illustrating the patient selecting process.

From January 2021 to December 2021, our hospital treated 169 patients with osteoporotic single-segment vertebral compression fractures, including 142 females and 27 males. Among these, 150 vertebrae met the inclusion criteria, involving 127 females and 23 males (**Figure 1**). All patients had complete preoperative bone density, CT, and MRI data, as well as postoperative X-rays. If postoperative X-rays failed to determine the presence of leakage, a CT scan would be performed. All preoperative and postoperative imaging examinations for the patients were conducted using the same scanner at our hospital. This study was approved by the Institutional Review Board.

The inclusion criteria included: 1) Severe back pain with limited mobility, which did not improve after systematic conservative treatment; 2) Vertebral compression fracture or vertebral bone marrow edema at the site of pain observed using MRI; and 3) Dual-energy X-ray absorptiometry showing a T-score ≤ -2.5.

The exclusion criteria included: 1) Vertebral fracture with infection or uncontrolled systemic infection; 2) Vertebral compression degree of Genant’s semi-quantitative (GSQ) grade 2 or higher; 3) Vertebral body undergoing pathological examination; or 4) Patients with severe medical conditions who could not tolerate surgery or presented contraindications for surgery.

### 2.2 Research methods

#### 2.2.1 Surgical method

After anesthesia, the patient was placed in a prone position. Under C-arm fluoroscopy, the affected vertebra was located and marked, and a 5 mm incision was made about 5-10 mm lateral to the pedicle projection point on both sides of the affected vertebra based on the C-arm fluoroscopy results. Then, a puncture needle was inserted, with the needle tip positioned at the 10 o’clock and 2 o’clock directions on the upper outer edge of the pedicle root, at an inward inclination angle of about 10-15°. Subsequently, the puncture needle was carefully inserted, ensuring that under anteroposterior fluoroscopy, the needle tip aligned with the inner edge of the pedicle root. Simultaneously, under lateral fluoroscopy, the needle tip was positioned precisely at the posterior edge of the vertebral body. The puncture needle was hammered until its tip was about 3 mm in front of the posterior edge of the vertebral body. Subsequently, the needle core was removed, and a hand drill was inserted along the working cannula to create a working channel, reaching approximately 3-5 mm from the anterior edge of the vertebral body. Then, the hand drill was removed. A balloon was inserted into each working channel, and inflated with a contrast agent under fluoroscopic guidance. The balloon was deflated and removed after 2 minutes and 30 seconds. Under C-arm fluoroscopy, bone cement in a paste-like consistency (about 3 minutes after preparation) was injected until satisfactory filling was achieved. After the cement was dried and hardened sufficiently, the push tube was rotated to ensure complete separation from the bone cement before it solidified. The volume of injected bone cement was documented, and the incision site was compressed to control bleeding before being dressed with sterile materials, marking the conclusion of the surgery.

#### 2.2.2 Radiological analysis

**Figure 2:**
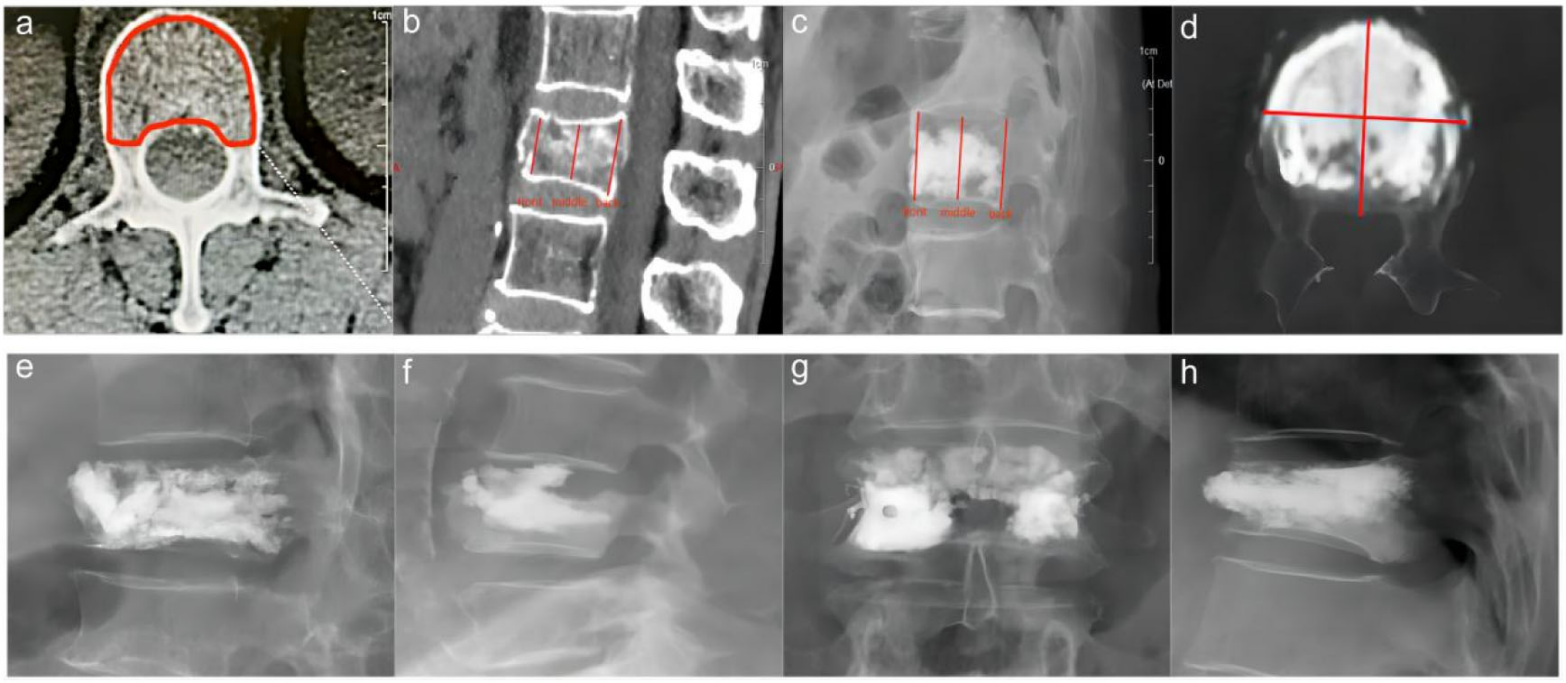
Preoperative and postoperative radiographic images of the fractured vertebra and the site of cement leakage. **(A)** Example of vertebral body cross-sectional area measurement. **(B)** Example of preoperative vertebral height measurement. **(C)** Example of postoperative vertebral height measurement. **(D)** Example of CT cross-sectional scan showing a large amount of cement distribution in all four quadrants of the vertebral body. **(E)** Example of bone cement leakage to the posterior vertebra; **(F)** Example of bone cement leakage into the intervertebral disc. **(G)** Example of bone cement leakage to the paravertebral vein. **(H)** Example of bone cement leakage to anterior vertebra.

The miPlatform Viewer51 software was used to calculate the vertebral volume: The patient’s CT data were imported into the miPlatform Viewer51 software. At a level near the center of the vertebral body, the cross-sectional area and height of the vertebral body (average of the heights at the anterior, middle, and posterior edges of the vertebral body) were measured. The vertebral volume was then calculated as the vertebral volume = vertebral cross-sectional area × vertebral height (**Figures 2A, B**). Then, the volume of each vertebral body was measured by three spine surgeons and radiologists, with the average value taken as the volume of that vertebral body, accurate to 0.01 cm³. Postoperative follow-up involved X-ray and CT examinations of the affected vertebral body to assess the restoration of vertebral height and Cobb angle, as well as the distribution of bone cement (**Figures 2C, D**). Two resident physicians jointly observed and identified any bone cement leakage and the specific location of the leakage (**Figures 2E–H**). The bone cement filling ratio was calculated following the formula: bone cement filling ratio = (injected bone cement volume/preoperative vertebral body volume) × 100%.

#### 2.2.3 Grouping basis

**Figure 3:**
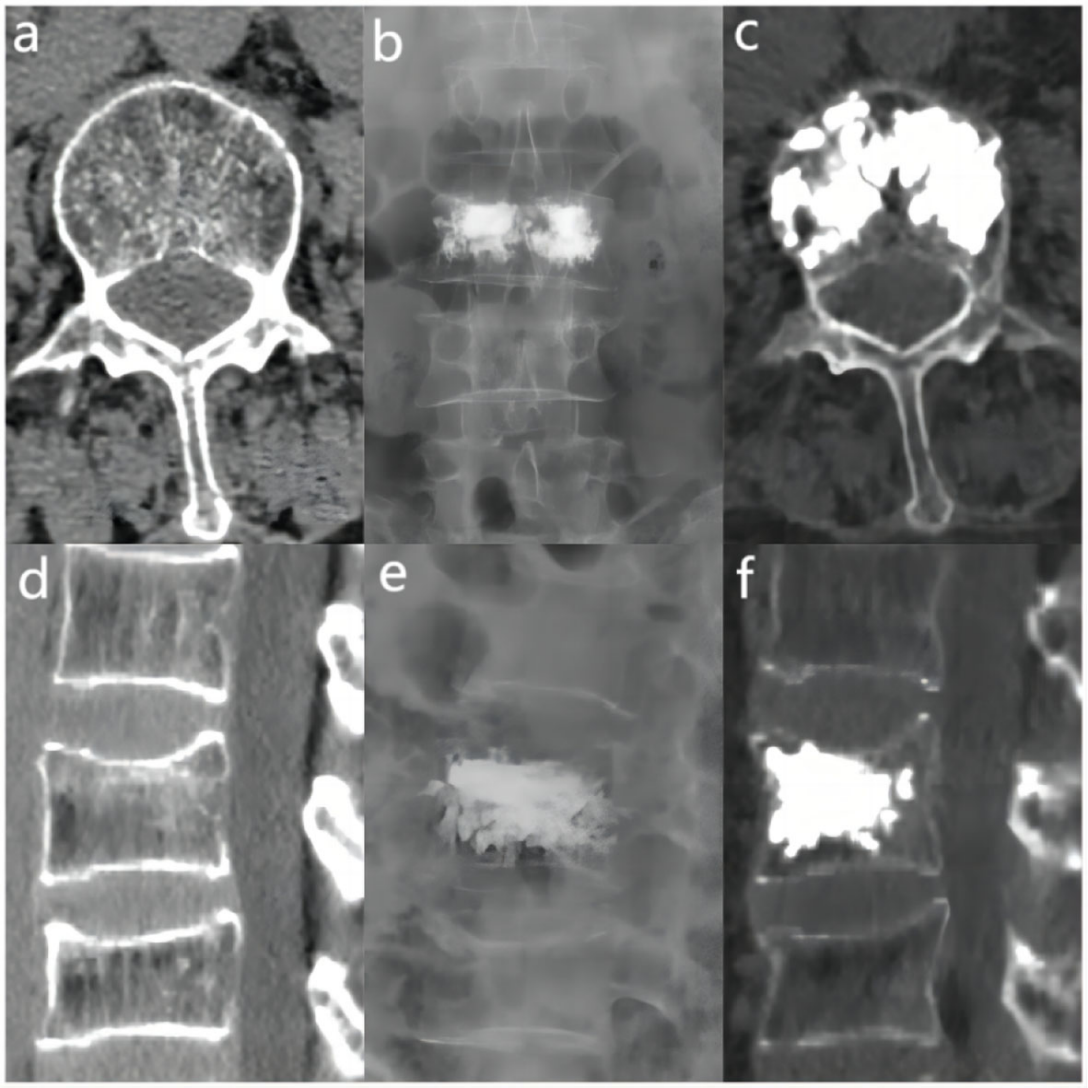
In the “< 0.4” cement infusion proportion group, a 67-year-old female was diagnosed with L1 osteoporotic vertebral compression fracture (OVCF). **(A)** CT cross-sectional image of the L1 vertebral body. **(B)** Postoperative ortho-X-ray examination of the lumbar spine showing sufficient distribution of bone cement in the vertebra. **(C)** CT cross-sectional scan showing a large distribution of bone cement in the vertebra. **(D)** Sagittal CT view of the L1 vertebral body. **(E)** Postoperative lateral X-ray examination of the L1 vertebral body. **(F)** Sagittal CT scan showing the distribution of bone cement in the vertebral body.

**Figure 4:**
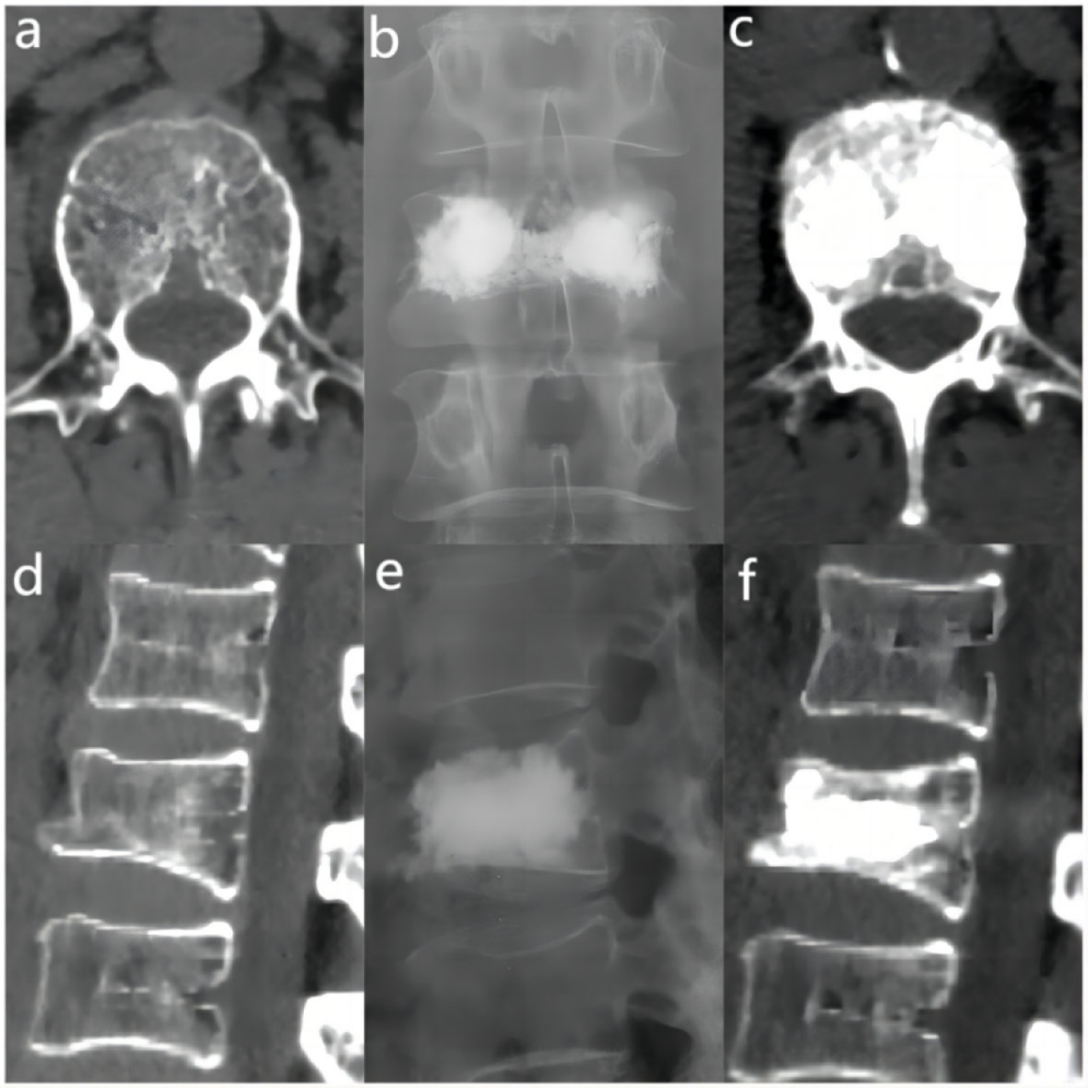
In the “0.4 - 0.6” cement infusion proportion group, a 71-year-old female was diagnosed with L2 osteoporotic vertebral compression fracture (OVCF). **(A)** CT cross-sectional image of the L2 vertebral body. **(B)** Postoperative ortho-X-ray examination of the lumbar spine showing sufficient distribution of bone cement in the vertebra. **(C)** CT cross-sectional scan showing a large distribution of bone cement in the vertebra; **(D)** Sagittal CT view of the L2 vertebral body. **(E)** Postoperative lateral X-ray examination of the L2 vertebral body. **(F)** Sagittal CT scan showing the distribution of bone cement in the vertebral body.

**Figure 5:**
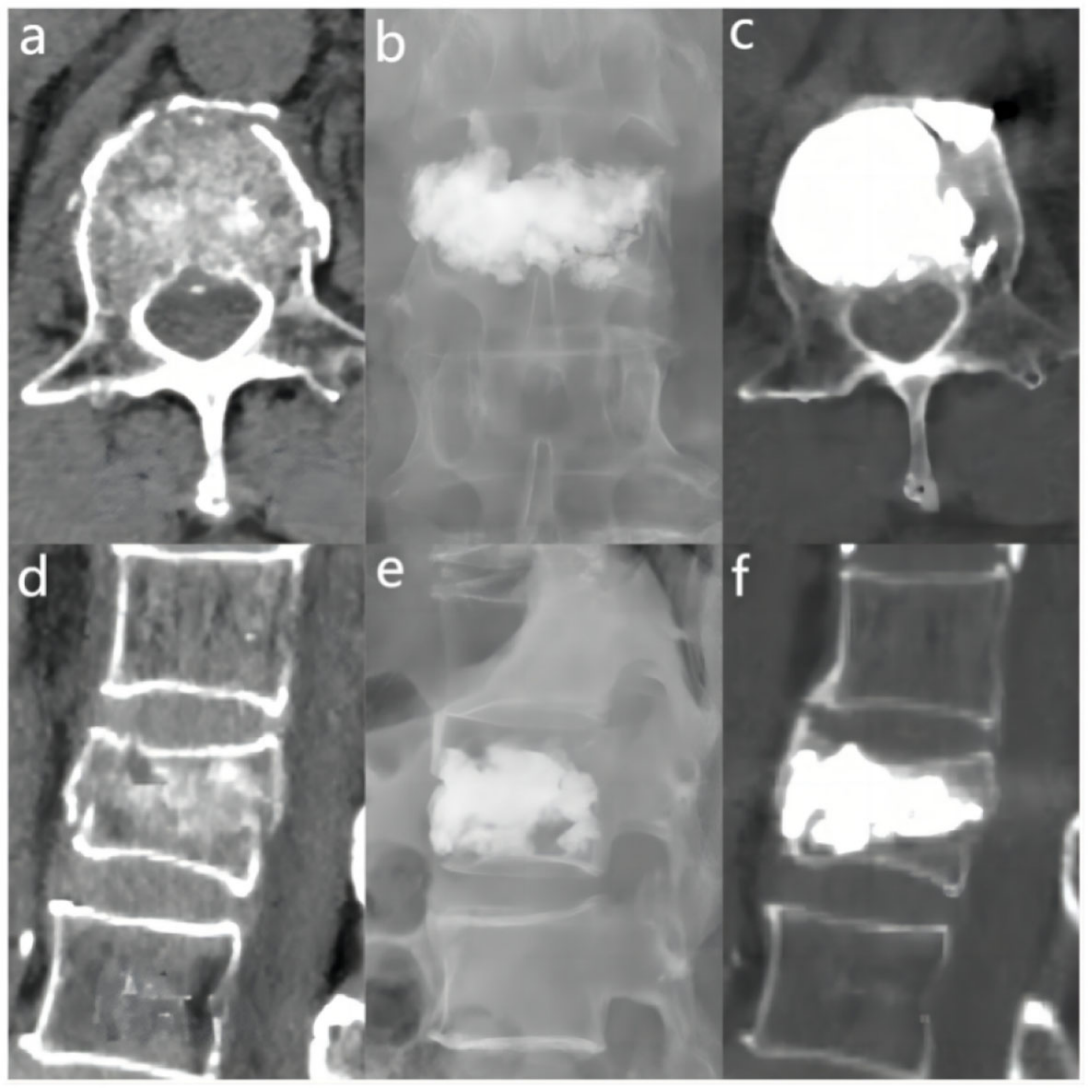
In the “> 0.6” cement infusion proportion group, a 72-year-old male was diagnosed with L1 osteoporotic vertebral compression fracture (OVCF) after a fall. **(A)** CT cross-sectional image of the L1 vertebral body. **(B)** Postoperative ortho-X-ray examination of the lumbar spine showing sufficient distribution of bone cement in the vertebra. **(C)** CT cross-sectional scan showing a large distribution of bone cement in the vertebra. **(D)** Sagittal CT view of the L1 vertebral body. **(E)** Postoperative lateral X-ray examination of the L1 vertebral body. **(F)** Sagittal CT scan showing the distribution of bone cement in the vertebral body.

The bone cement filling ratio was hereby adopted as the basis for grouping. Compared to other studies grouping patients based on the volume of bone cement injected, the proposed approach offered a distinct advantage. It recognized that patients varied in height and weight, resulting in differing vertebral volumes. Therefore, utilizing the volume of bone cement in milliliters for grouping might lack rationality and individualization. Instead, representing the injected bone cement amount as a percentage of the post-fracture vertebral volume proved to be a more logical and precise approach. Based on existing literature and clinical experience, the bone cement filling ratios were hereby divided into the “<0.4” group (**Figure 3**), the “0.4-0.6” group (**Figure 4**), and the “>0.6” group (**Figure 5**).

#### 2.2.4 Statistical analysis methods

Statistical analysis was conducted using SPSS 25.0 software. Measurement data following a normal distribution were represented by `x ± s, while non-normally distributed data were denoted by M (Q₁, Q₃). For the statistical comparison of cement leakage among the three groups, Fisher’s Exact Test was carried out. Postoperative outcome categorical variables were compared using the Chi-square test. Normally distributed continuous variables with homogeneity of variance were subjected to analysis using Analysis of Variance (ANOVA). Conversely, variables that did not adhere to a normal distribution were evaluated utilizing the Kruskal-Wallis H test. A p-value < 0.05 was considered statistically significant.

## 3 Results

### 3.1 Outcomes and complications of vertebral bone cement distribution after PKP surgery

**Figure 6:**
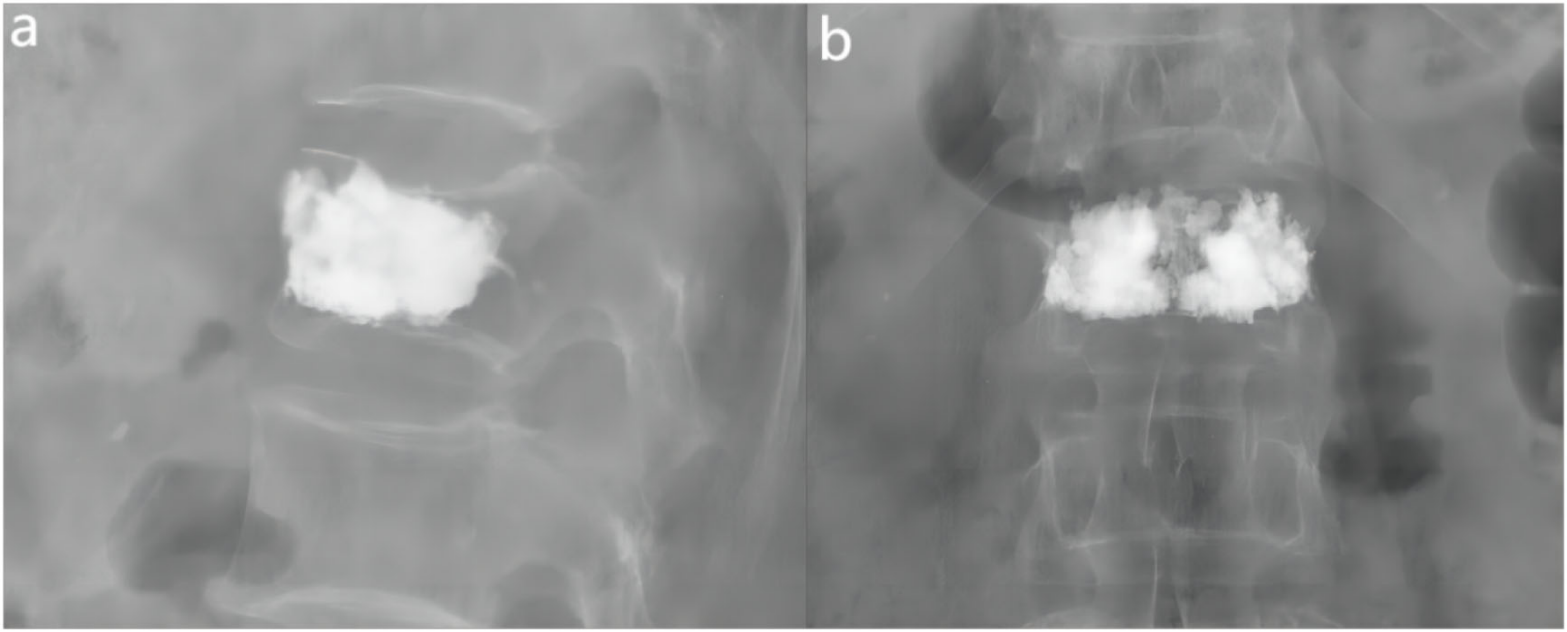
Examples of anterior-lateral radiographs after surgery for fractured vertebrae. **(A)** The lateral X-ray view showed that the distribution of bone cement reached the anterior and posterior edges of the vertebral body. **(B)** The ortho-X-ray view showed the distribution of bone cement near the upper and lower endplates.

In this clinical study, all patients were systematically followed up after surgery at the specific time points of the second postoperative day, one month postoperatively and three months postoperatively. Imaging follow-up showed that the distribution of cement in the vertebral body was close to the right and left margins and the upper and lower endplates by analysis of postoperative X-ray orthopantomograms and lateral radiographs; in lateral radiographs, the distribution of cement was close to the upper and lower endplates and reached the anterior and posterior margins of the vertebral body anteriorly and posteriorly (**Figures 6A, B**). In addition, the homogeneous distribution of large amounts of bone cement in all four quadrants of the vertebral body was further verified by CT cross-sectional scanning (**Figure 2D**). There were 57 cases of cement leakage, of which, 34 involved intradiscal leakage, including 8 of combined leakage from other sites. There were 13 cases of cement leakage to the paravertebral veins and a total of 8 cases of leakage to the anterior and lateral vertebral body. Meanwhile, 2 cases of combined posterior vertebral body cement leakage were observed, both in the “>0.6” group, but no serious complications such as spinal cord compression or distal vascular embolism occurred. No cases of re-fracture were detected. Notably, 1 case of adjacent vertebral fracture occurred within 1 week after surgery, and the patient’s pain symptoms were effectively relieved after retreatment with PKP. Overall, these results demonstrated that while cement leakage was common, it did not cause major complications in this study. Most patients experienced significant pain relief after PKP treatment.

### 3.2 Univariate analysis of baseline characteristics

In order to present detailed statistical data on various clinical measures related to the cement ratio, a one-way analysis of baseline characteristics was generated for this study (**Table 1**). When comparing the clinical data on the percentage of cement infusion in the three groups, the differences were founded to be statistically significant in terms of cement leakage, three-month postoperative Visual analogue scale (VAS), postoperative oswestry disability index (ODI), and the degree of vertebral height restoration (p-value < 0.05 in all cases).

**Table 1:** Univariate analysis of baseline characteristics.

Variables	Total (n = 150)	Cement Proportion Group <0.4 (n = 17)	Cement Proportion Group >0.6 (n = 61)	Cement Proportion Group 0.4-0.6 (n = 72)	Statistic	Statistical significance
Age, years	71.847 ± 9.292	74.118 ± 9.636	70.836 ± 9.869	72.167 ± 8.702	F=0.910	0.405
Preoperative Vertebral Height, cm	1.772 ± 0.334	1.904 ± 0.335	1.619 ± 0.350	1.869 ± 0.266	F=12.489	<.001
BMD, g/cm²	0.687 ± 0.139	0.775 ± 0.086	0.667 ± 0.120	0.682 ± 0.156	F=4.250	0.016
L1 Hounsfield unit, HU	63.530 ± 35.491	58.032 ± 34.844	63.984 ± 35.694	64.443 ± 35.845	F=0.230	0.795
BMI, kg/m2	23.438 (21.259, 25.450)	22.491 (21.453,27.055)	23.243 (21.231,24.655)	23.730 (21.405,25.942)	χ²=1.286#	0.526
Preoperative Cobb Angle, °	12.300 (6.875, 19.725)	10.400 (6.300,13.600)	13.400 (8.700,19.800)	12.950 (6.250,20.075)	χ²=1.713#	0.425
Postoperative Cobb Angle, °	11.300 (5.425, 16.250)	8.800 (3.600,12.900)	12.000 (6.200,16.600)	10.400 (4.500,16.300)	χ²=2.120#	0.346
Postoperative Vertebral Height, cm	2.358 (2.003, 2.565)	2.410 (2.023,2.530)	2.200 (1.820,2.510)	2.398 (2.221,2.628)	χ²=9.534#	0.009
Pre-op Oswestry Disability Index, (0–100%)	86.667 (82.222, 88.889)	86.667 (84.444,88.889)	86.667 (82.222,86.667)	84.444 (82.222,88.889)	χ²=0.704#	0.703
Oswestryscore Index 2nd Day Post-op, (0–100%)	26.667 (24.444, 28.889)	24.444 (24.444,26.667)	26.667 (24.444,28.889)	26.667 (24.444,26.667)	χ²=12.962#	0.002
Oswestryscore Index 1 Month Post-op, (0–100%)	20.000 (17.778, 20.000)	20.000 (17.778,22.222)	20.000 (20.000,24.444)	17.778 (17.778,20.000)	χ²=37.296#	<.001
Oswestryscore Index 3 Month Post-op, (0–100%)	13.333 (11.111, 15.556)	13.333 (11.111,17.778)	15.556 (13.333,15.556)	11.111 (11.111,13.333)	χ²=29.279#	<.001
T-score	-3.100 (-3.900, -2.400)	-2.800 (-3.000,-2.600)	-3.100 (-4.100,-2.300)	-3.100 (-3.900,-2.400)	χ²=2.774#	0.250
Z-score	-1.100 (-1.900, -0.425)	-1.600 (-1.700,-0.900)	-1.100 (-2.000,-0.300)	-0.950 (-2.100,-0.375)	χ²=0.400#	0.819
Pre-op VAS Score, (0–10)	7.000 (7.000, 8.000)	7.000 (7.000,8.000)	7.000 (7.000,8.000)	7.000 (7.000,8.000)	χ²=0.125#	0.940
VAS score 2nd Day Post-op, (0–10)	2.000 (1.000, 2.000)	2.000 (2.000,2.000)	2.000 (1.000,2.000)	2.000 (1.000,2.000)	χ²=2.917#	0.233
VAS score 1 Month Post-op, (0–10)	1.000 (0.000, 1.000)	1.000 (1.000,1.000)	1.000 (0.000,1.000)	1.000 (0.000,1.000)	χ²=3.881#	0.144
VAS score 3 Month Post-op, (0–10)	0.000 (0.000, 0.000)	0.000 (0.000,1.000)	0.000 (0.000,1.000)	0.000 (0.000,0.000)	χ²=12.322#	0.002
Gender, F/M					χ²=6.032	0.049
Female	127 (84.667)	11 (64.706)	54 (88.525)	62 (86.111)		
Male	23 (15.333)	6 (35.294)	7 (11.475)	10 (13.889)		
Genant’s Semi-quantitative Method					χ²=4.398	0.111
Grade 0	46 (30.667)	7 (41.176)	13 (21.311)	26 (36.111)		
Grade 1	104 (69.333)	10 (58.824)	48 (78.689)	46 (63.889)		
Kümmell’s Disease					χ²=3.913	0.141
No	123 (82.000)	11 (64.706)	51 (83.607)	61 (84.722)		
Yes	27 (18.000)	6 (35.294)	10 (16.393)	11 (15.278)		
Osteoporosis therapy					χ²=13.009	0.001
Treatment with oral calcium supplements and calcitriol only	106 (70.667)	6 (35.294)	49 (80.328)	51 (70.833)		
Treatment with oral calcium supplements and calcitriol, combined with intravenous administration of ibandronate or zoledronic acid	44 (29.333)	11 (64.706)	12 (19.672)	21 (29.167)		
Cement Leakage					χ²=10.650	0.005
No	93 (62.000)	10 (58.824)	29 (47.541)	54 (75.000)		
Yes	57 (38.000)	7 (41.176)	32 (52.459)	18 (25.000)		

Values are shown as mean  ±  SD, M (Q₁, Q₃) or n (%).

F: ANOVA, #: Kruskal-waills test, χ²: Chi-square test, -: Fisher exact.

SD, standard deviation; M, Median; Q₁, 1st Quartile; Q₃, 3st Quartile.

### 3.3 Low cement leakage of the “0.4-0.6” group

By applying Fisher’s Exact Test to compare the leakage of the three different cement percentage groups, the results showed that “<0.4” *vs*. “0.4-0.6” of the two groups of (p=0.232), “<0.4” *vs*. “>0.6” of the two groups of (p=0.584), indicating that there was statistically no cement leakage between these two groups significant difference. For the comparison of the “0.4-0.6” versus “>0.6” groups (p=0.0013), this indicated a statistically significant difference in the rate of cement leakage between these two groups.

**Figure 7:**
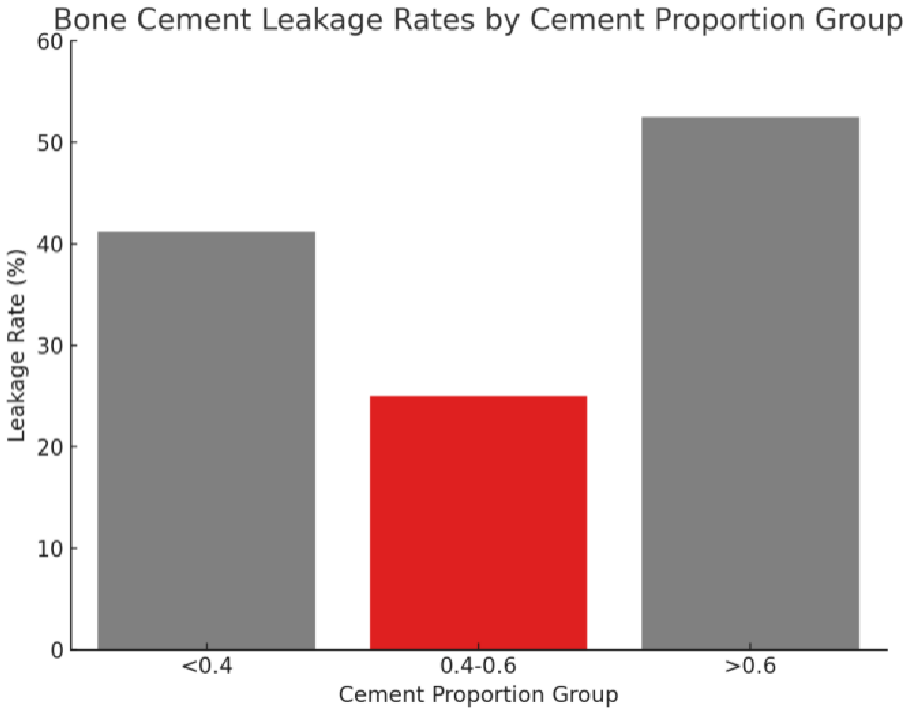
The leakage rate of bone cement in different proportion groups. The figure presents three distinct cement proportion groups: “<0.4,” “0.4-0.6,” and “>0.6.” The bar chart indicates the leakage rate for each group, with the “0.4-0.6” group displaying the lowest rate of bone cement leakage.

Therefore, based on the results of Fisher’s Exact Test, the conclusion was drawn that there was a significant difference in cement leakage rate between only the “0.4-0.6” and “>0.6” groups. The leakage rates for the three different cement percentage groups of “<0.4”, “0.4-0.6”, and “>0.6” were 41.18%, 25.00%, and 52.46%, respectively (**Figure 7**). Based on these findings, the “0.4-0.6” group was found to present the lowest cement leakage rate among the three groups. This finding had important clinical implications for reducing the risk of postoperative complications. Therefore, the present study advocated for careful consideration of ratio selection in bone cement application. A preferred cement ratio of 0.4-0.6 was recommended in practice to mitigate the risk of leakage, ultimately enhancing postoperative outcomes and improving patient quality of life.

### 3.4 An optimal cement ratio of 0.4-0.6 for postoperative pain relief and functional recovery

**Figure 8:**
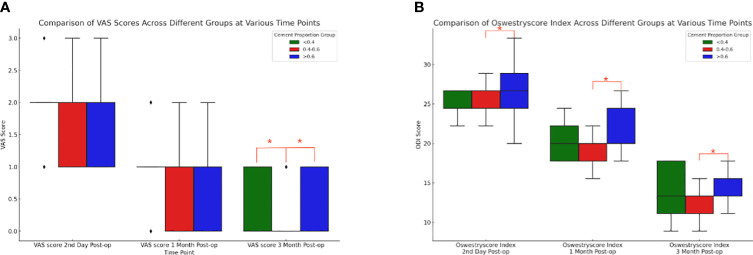
Comparison of box plots of Visual Analog Scale (VAS) and Oswestry Disability Index (ODI) scores for cement percentage between the three groups. **(A)** VAS (Visual Analog Scale) scores at three postoperative time points for the three groups with different proportions of bone cement. At three months post-surgery, there were notable differences between the “<0.4” group and the “0.4-0.6” group, as well as between the “0.4-0.6” group and the “>0.6” group. The “0.4-0.6” group had significantly lower VAS scores compared to the “>0.6” group, indicating that the “0.4-0.6” group experienced better pain improvement. **(B)** For patients in the three groups with different bone cement proportions, there were statistically significant differences in the ODI (Oswestry Disability Index) scores between the “0.4-0.6” group and the “>0.6” group at three postoperative time points. Compared to the “>0.6” group, the “0.4-0.6” group showed more significant improvement on the second day, one month, and three months post-surgery, indicating better functional recovery.

Based on the results of the Shapiro-Wilk test, the Kruskal-Wallis H test was further conducted to compare the differences in VAS and ODI preoperatively and at three postoperative time points between three different cement ratio subgroups (“<0.4”, “0.4-0.6”, and “>0.6”). The study results showed no statistically significant difference between preoperative VAS (p=0.125), postoperative day VAS (p=0.233), postoperative one month VAS (p=0.144), and preoperative ODI (p=0.703). However, it should be noted that the differences in three months postoperative VAS (p=0.002), second day postoperative ODI (p=0.002), one month postoperative ODI (p<0.001), and three months postoperative ODI (p<0.001) were statistically significant. Meanwhile, two-by-two comparisons between different bone cement ratio groups were performed using the Mann-Whitney U test, while the Bonferroni correction was utilized to adjust the p-value for multiple comparisons. The corrected significance level was 0.017, indicating a significant difference in VAS at three months postoperatively between the three groups (**Figure 8A**), and “0.4-0.6” and “>0.6” at the second postoperative day, one month postoperatively, and three months postoperatively ODI (**Figure 8B**). From these results, it could be inferred that at three months postoperatively, the “0.4-0.6” group showed better pain improvement than the “>0.6” group. In terms of ODI, the “0.4-0.6” group presented significantly better improvement than the “>0.6” group on the second day, one month, and three months postoperatively, suggesting better functional recovery in the “0.4-0.6” group.

### 3.5 Comparison of the restoration degree of vertebral body height and the degree of restoration of the Cobb angle of the posterior convexity between groups

**Figure 9:**
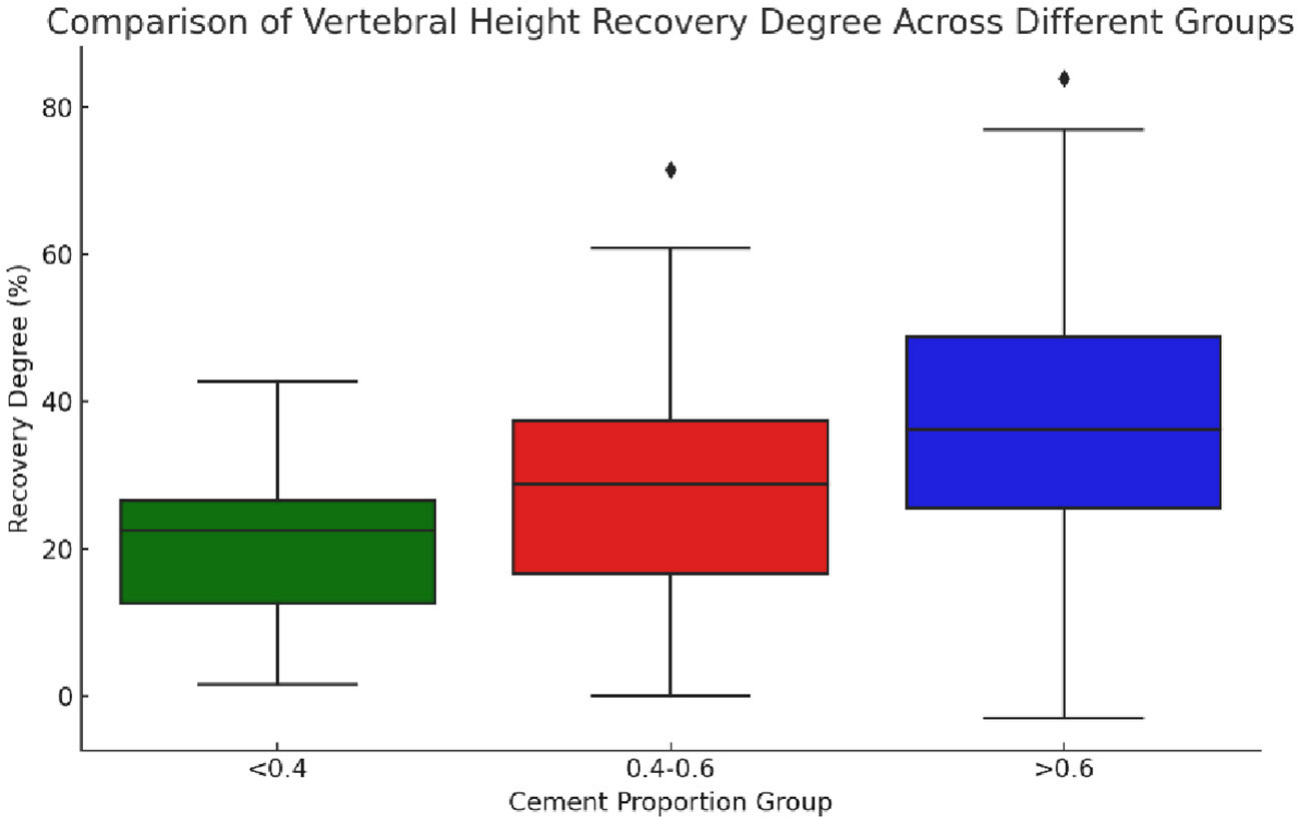
Boxplot of vertebral height restoration among three groups with different bone cement infusion proportions. The figure showed that the median vertebral height restoration in the “>0.6” group was the highest, indicating that the median patient in this group experienced better vertebral height restoration. The “0.4-0.6” group followed in terms of restoration, while the “<0.4” group had the lowest level of restoration. The “>0.6” group had the largest IQR (interquartile range), suggesting that the variability in restoration levels was also the greatest in this group.

The statistical data of the preoperative and postoperative vertebral heights of the groups showed a significant difference (χ²=9.534, p=0.009), indicating that at least one group was significantly different from the others in terms of vertebral height recovery. The recovery degree was calculated following the following formula: degree of recovery = postoperative vertebral height - preoperative vertebral height preoperative vertebral height x 100%. Postoperative heights did not conform to a normal distribution, and the degree of recovery was calculated based on changes in preoperative and postoperative heights. Actually, the median and IQR should be more appropriate for comparison. The median and interquartile range (IQR) of the recovery degree of vertebral height in each group were as follows: the “0.4-0.6” group: median degree of recovery: 28.74%, and IQR degree of recovery: 20.85%; the “<0.4” group: median degree of recovery: 22.47%, and IQR degree of recovery: 13.89%; and the “>0.6” group: median degree of recovery: 36.20%, and IQR degree of recovery: 23.13% (**Figure 9**).

Comparison of medians showed that the “>0.6” group had the highest median degree of vertebral height recovery, implying that the median patient in this group had better vertebral height recovery, followed by the “0.4-0.6” group. Besides, the “<0.4” group had the lowest degree of recovery. The IQRs provided information about the distribution of the data, with larger IQRs suggesting greater within-group variability in the degree of recovery. Herein, the “>0.6” group had the largest IQR, indicating the greatest variability in the degree of recovery.

These results provided a more comprehensive perspective to compare the degree of vertebral height recovery in different groups, taking into account central tendency and variability in data distribution. Overall, in terms of vertebral height recovery, the “>0.6” group performed best while presenting the greatest variability in the degree of recovery.

Cobb angle changes in the “<0.4”, “0.4-0.6” and “>0.6” groups (p=0.9663) indicated no statistically significant difference between the groups.

## 4 Discussion

As the population ages, the number of osteoporosis patients is steadily rising each year (4). Causes of osteoporotic fractures include minor trauma, sneezing, coughing, etc. in daily life (14). Treatment of osteoporotic fracture includes conservative treatment and surgery, while conservative treatment includes bed rest, physical therapy, medication and other methods (15). However, conservative treatment options for osteoporosis can inadvertently lead to complications. Prolonged bed rest, for instance, can exacerbate osteoporosis by further weakening bones. Additionally, it can increase the risk of urinary tract infections and lower extremity deep vein thrombosis, etc. (16). PKP is a minimally invasive procedure yielding satisfactory results in the treatment of osteoporotic compression fractures of the vertebral body (17). Postoperative complications of PKP, such as re-fracture of the injured vertebrae, cement leakage, and fracture of the adjacent vertebrae, are related to the amount and distribution of intraoperative cement infusion (18). Most current studies adopt the number of milliliters of bone cement instilled in the fractured vertebrae as a measure of the amount of bone cement instilled (19). It is generally acknowledged that vertebral body size varies among different races, genders, and sites, making it more reasonable to express the degree of vertebral body perfusion by the ratio of cement infusion and vertebral body volume.

Previous studies have shown that in the treatment of osteoporotic compression fractures of the vertebral body, a small amount of bone cement infusion can significantly alleviate the pain symptoms of the patients. When the volume ratio of bone cement infusion reaches 0.15-0.3, the initial stiffness of the diseased vertebrae can be restored, and a satisfactory therapeutic effect can be obtained (20–22). However, insufficient cement infusion and uneven distribution can weaken the vertebral body, failing to restore it to its preoperative strength. This increases the risk of vertebral re-fracture events (10, 23). To prevent recurrence of the fracture, bone cement should be injected to increase the strength of the vertebral body above the pre-injury level whenever possible (21). Ideal cement distribution aims to fill the injured vertebrae as much as possible. On imaging, the cement should extend to the upper and lower endplates of the injured vertebra, as well as cover its anterior and posterior margins, along with the right and left lateral margins of the vertebral body (24, 25). It has been shown that the strength of the vertebral body increases 2-fold when the bone cement contacts one side of the vertebral body endplate, while the strength of the vertebral body can increase 12-fold when the bone cement contacts the upper and lower endplates at the same time. In this case, the vertebral body is effectively prevented from collapsing, and vertebral re-fracture of the patient’s body can be avoided (26).

However, too much bone cement infusion may excessively strengthen the diseased vertebrae and increase stress, leading to adjacent vertebral fractures (10, 23). At the same time, excessive bone cement infusion can result in leakage events. In severe instances, the bone cement entering the spinal canal might compress nerve tissue, potentially causing paralysis in the patient (27). Therefore, an appropriate proportion of cement infusion in PKP can yield good patient outcomes and reduce the incidence of clinical adverse events. Herein, clinical characteristics and follow-up data of 150 patients with vertebral fractures who underwent PKP were collected, and it was concluded through statistical analysis that in PKP, a cement infusion ratio of 0.4-0.6 ensured that the vertebral body of the patient was satisfactorily filled, that the distribution of the cement was relatively homogeneous, that leakage did not occur because of too much cement infusion, and that a balance was achieved between reducing complications and good patient prognosis. This amount of cementing achieved a satisfactory balance, minimizing complications while ensuring a positive prognosis for the patient.

In this study, the incidences of cement leakage events between three different cement infusion ratio groups was investigated, which were 41.18%, 25% and 52.46% for the three different cement ratio groups of “<0.4”, “0.4-0.6” and “>0.6”, respectively. Among them, the reason for the higher incidence of leakage events in the cement infusion ratio “<0.4” group might be closely related to Kümmell’s Disease. In the present study, 35% of the patients in the “<0.4” group had Kümmell’s disease, a spinal disease associated with osteoporosis, in which the internal structure of the vertebral body was often altered due to the effects of the disease, such as the presence of cavities in the vertebral body or unhealed fractures (28). A low percentage of bone cement infusion might result in the bone cement not being evenly distributed during injection, increasing the risk of leakage (29). In cases where the vertebral body is damaged or weakened by Kümmell’s Disease, the anterior and posterior columns of the vertebral body experience distinct mechanical stresses. This imbalance in internal pressure distribution can contribute to cement leakage toward regions of lower pressure (30). In addition, in the “>0.6” group in this study, 32 cement leakage events were counted, of which intradiscal leakage occurred in 20 cases. Among these 20 cases, 3 were combined with leakage from other sites, but no serious complications such as spinal cord compression or distal vascular embolism were observed. The high incidence of intradiscal leakage events was closely associated with fractures of the upper and lower endplates of the vertebral body, and unhealed fracture lines might provide a direct escape route for highly infused bone cement, leading to injury to adjacent vertebrae or increasing the risk of new compression fractures (31). The cement infusion ratio of the “0.4-0.6” group had the lowest incidence of leakage events among the three groups, and it was predicted that cement infusion in this range reduced the incidence of adverse clinical events and improved the postoperative quality of life of patients with vertebral fractures.

VAS and ODI are key tools for evaluating pain and function in postoperative patients, vital for tracking the recovery of those with fractures. They help assess treatment efficacy and inform future treatment strategies (32). In this study, 150 patients with vertebral fractures who underwent percutaneous vertebral kyphoplasty were followed up preoperatively, at day 2, 1 month and 3 months postoperatively. The follow-up data were statistically analyzed. The results of the analysis showed that at three months postoperatively, the VAS of the “0.4-0.6” group was significantly lower than that of the remaining two groups, and the patients’ pain symptoms were considerably improved. Regarding the patients’ postoperative functional recovery, the ODIs of patients with a bone cement filling ratio of 0.4-0.6 at the 2nd day, 1 month and 3 months after surgery were statistically significant compared with those of patients with a bone cement filling ratio of >0.6. This suggested that patients with a bone cement filling ratio of 0.4-0.6 had a better postoperative functional recovery. Besides, the toxic effect of bone cement and the high temperature reaction produced during the curing process destroyed the nerves and blood vessels of the diseased vertebrae, which could reduce the pain symptoms of the patients and help recover their functions after surgery (33). In those patients with low cement perfusion ratios, higher postoperative VAS and ODI might be associated with a lower amount of cement that could lead to underfilling, with the injected cement only acting as a support rather than curing the fractured vertebrae (20). In addition, the higher modulus of elasticity of hardened bone cement compared to osteoporotic vertebrae increased the likelihood of fracture recurrence, significantly impacting postoperative functional recovery and potentially inducing new pain symptoms in patients (34). Several studies have also shown a positive correlation between improvement in VAS and ODI as the amount of cement injected in a given area increases (35, 36). However, excessive cement infusion ratios can lead to poor outcomes due to leakage events. Depending on the site, these can cause serious postoperative complications like fractures of neighboring vertebrae, upper and lower endplate injuries, and pulmonary embolism, impacting the patient’s functional recovery, inducing new pain symptoms, and even endangering their life (12, 37).

The degree of postoperative vertebral body recovery in patients with vertebral body fracture has an important impact on the patients’ postoperative prognosis. Better vertebral body height recovery can reduce vertebral body instability and improve the biomechanical status of the spine, thus reducing pain and improving the patients’ quality of life (38, 39). In the present study, the “0.4-0.6” group showed a higher median vertebral height recovery and a relatively narrow interquartile range (IQR) in this group, indicating a relatively centralized recovery effect with less variability. This possibly implied that within this interval of the perfusion ratio, patients recovered with greater consistency and lower risk. In addition, the group with a cement perfusion ratio above 0.6 displayed a higher upper recovery limit, but with a wider IQR, signaling increased uncertainty and potentially greater complication risks. Opting for a ratio between 0.4 and 0.6 could strike a balance, ensuring effective vertebral height recovery while minimizing both risk and variability.

In this study, only mild to moderate vertebral compression cases were included due to concerns about calculation errors. Severe compression fractures could cause substantial changes in vertebral volume after balloon dilatation, impacting result accuracy. During surgery, a conservative restoration strategy was adopted to avoid excessive restoration and vertebral body stress. This approach might help reduce the risk of vertebral body re-fracture and adjacent vertebral body fracture. Patients undergoing postoperative anti-osteoporotic therapy were also monitored, revealing fewer incidents of vertebral body re-fracture and a reduced occurrence of adjacent vertebral body fractures. Only one adjacent vertebral body fracture was recorded, a notably lower rate compared to findings in other studies (40). This outcome might be attributed to the surgical approach and the implementation of aggressive postoperative anti-osteoporotic therapy.

While this study offered important insights regarding cement perfusion ratios in percutaneous vertebral kyphoplasty, particularly emphasizing a perfusion ratio of 0.4-0.6 as a balance between efficacy and safety, there were still some limitations to the analysis. First, the results might not be broadly representative of all vertebral fracture patients due to the single-center study and small sample size. Furthermore, the homogeneity of the sample limited the observation of possible differences in response to treatment among different populations. Future studies should include more diverse patient populations, including patients of different ethnicities, cultural backgrounds, and geographic locations, and expand to multiple centers to enhance the generalizability of the findings. In addition, the follow-up duration might have captured all potential long-term treatment effects and complications. Longer follow-up durations and larger sample sizes could improve the accuracy of treatment recommendations, ensuring that the patients could receive personalized and optimized treatment plans in clinical practice.

## 5 Conclusion

In percutaneous vertebral kyphoplasty for the treatment of osteoporotic compression fractures, 0.4-0.6 serves as an appropriate cement infusion ratio to achieve better results between reduced complications and good patient prognosis.

## Data availability statement

The original contributions presented in the study are included in the article/supplementary material. Further inquiries can be directed to the corresponding author.

## Ethics statement

The studies involving humans were approved by The First Affiliated Hospital of Dalian Medical University. The studies were conducted in accordance with the local legislation and institutional requirements. The participants provided their written informed consent to participate in this study. Written informed consent was obtained from the individual(s) for the publication of any potentially identifiable images or data included in this article.

## Author contributions

NS: Writing – original draft, Writing – review & editing. YZ: Writing – review & editing, Data curation, Methodology, Supervision, Formal analysis, Validation, Investigation, Resources. DX: Writing – original draft, Writing – review & editing. YC: Writing – review & editing, Methodology, Supervision, Conceptualization, Software. YL: Writing – original draft, Writing – review & editing.
